# The Potential of Self-Assessment and Associated Factors for Delayed Symptomatic Hyponatremia Following Transsphenoidal Surgery: A Single Center Experience

**DOI:** 10.3390/jcm12010306

**Published:** 2022-12-30

**Authors:** Pia Roser, Klaus Christian Mende, Georgios K. Dimitriadis, Marius Marc-Daniel Mader, Jens Aberle, Jörg Flitsch, Roman Rotermund

**Affiliations:** 1 Department of Endocrinology and Diabetes, University Medical Center Hamburg Eppendorf, 20251 Hamburg, Germany; 2 Department of Neurosurgery, Friedrich-Ebert-Krankenhaus, 24534 Neumuenster, Germany; 3 Department of Endocrinology ASO/EASO COM, King’s College Hospital NHS Foundation Trust, Denmark Hill, London SE5 9RS, UK; 4 Department of Neurosurgery, University Hospital Hamburg-Eppendorf, 20251 Hamburg, Germany

**Keywords:** self-assessment, hyponatremia, pituitary, transsphenoidal surgery, SIADH

## Abstract

(1) Background: We identified screening parameters and associated factors for delayed, symptomatic hyponatremia (DSH) following inpatient discharge after transsphenoidal surgery (TSS). (2) Methods: In this prospective, monocentric study, 108 patients who underwent TSS for pituitary pathologies were included, provided with a questionnaire and instructed to document urine specific gravity, fluid intake/urine output, body weight and clinical symptoms for every of five days following discharge from hospital. (3) Results: The overall incidence of DSH within 14 days following discharge from the hospital was 14.8% (*n* = 9). Symptomatic patients presented on average 8.6 days after surgery. Mild DSH was present in 3.3% of the patients, moderate in 1.6% and severe hyponatremia in 9.8% of patients. Female sex (*p* = 0.02) and lower BMI (*p* = 0.02), as well as nausea (66.7%; *p* < 0.01) and emesis (33.3%; *p* < 0.05), were associated with DSH. A significant weight delta between morning and afternoon weight two days before the event of DSH between both groups (1.26 kg (*n* = 5) vs. 0.79 kg (*n* = 52), *p* < 0.05) was detected. (4) Conclusions: Handing out a symptom questionnaire at discharge seems to be an easy and feasible tool for the detection of DSH after hospital discharge.

## 1. Introduction

Electrolyte imbalances and dysregulation of fluid intake or urine output are possible complications of transsphenoidal surgery (TSS) for sellar pathologies. The incidence of hyponatremia, defined as a plasma sodium concentration <135 mmol/L, after TSS, has been reported to range between 2%–35% [1,2,3,4,5,6,7,8]. Undiagnosed hyponatremia is the leading cause of unplanned hospital readmissions within 30 days of TSS for pituitary tumours [9]. It can lead to major complications due to significant changes in sodium concentrations and fluid status with reported mortality rates up to 20% for patients with plasma sodium concentrations (Na) <120 mmol/L [10,11]. The two major causes of DSH are the syndrome of inappropriate antidiuretic hormone secretion (SIADH) and the cerebral salt-wasting syndrome (CSWS). As the onset of hyponatremia may often be delayed, diagnosis after discharge from the hospital is difficult [12]. In most cases, hyponatremia appears to occur either on the second to third postoperative day or at the eight to tenth postoperative day, leading to a biphasic course of manifestation [12]. This proves to be particularly problematic as most patients treated with TSS are discharged within five to eight days after surgery [13,14,15,16] and advances in surgical techniques over the next few years are expected to further shorten hospital stays. Different associated variables have been described in the past for the occurrence of hyponatremia, including tumour size, histopathology, Knosp grade for adenomas on MRI, parasellar invasion, operative approach, age, sex and involvement of the pituitary stalk [1,2,12,17,18]. However, we know that hyponatremia secondary to SIADH is still underdiagnosed and incorrectly managed among physicians [19]. Based on the hypothesis that there is a lack of awareness of the symptoms and occurrence of hyponatremia in patients undergoing TSS and that detection of especially severe hyponatremia, which in most of the cases is symptomatic, has to be improved, the aim of this prospective, single-center study was to find screening parameters for patients to self-detect symptomatic, postoperative hyponatremia after TSS and to seek for associated factors for hyponatremia with a standardized protocol including a symptom questionnaire.

## 2. Materials and Methods

### 2.1. Patient Eligibility

Between September 2016 and August 2018, one-hundred and eight patients treated with TSS for suprasellar and sellar pathologies at the Department of Neurosurgery of the University Medical Center Hamburg-Eppendorf, Germany, have been included in this study. None of the patients had revisional head surgery, radiotherapy or neoadjuvant medical treatments for functioning tumours. This study and the maintenance of a prospective database for pituitary surgery patients at the University Medical Center Hamburg-Eppendorf, was approved by the ethics committee of the Medical Association Hamburg (PV5589). All patients gave their informed, written consent. The conduct of this study was based on the regulations of the Declaration of Helsinki.

### 2.2. Standard Preoperative Evaluation and Inpatient Care

Preoperative data were collected following a standardized protocol for patients undergoing TSS at the Department of Neurosurgery of the University Medical Center Hamburg-Eppendorf. After measuring patients’ weight and height and calculating their Body Mass Index (BMI), laboratory assessments were performed including measurements of the pituitary hormone profile as well as electrolytes, full blood count and inflammatory markers. Preoperative imaging included thin-slice coronal and sagittal T1-weighted MRI of the sellar region with and without contrast enhancement using gadolinium. After TSS, vital signs, fluid intake and urine output were monitored and regular measurements of plasma sodium concentrations on postoperative day (POD) 1 and POD 3, as well as postoperative one-time assessment of the pituitary hormone profile, were performed in all patients.

### 2.3. Outpatient Care

At discharge, patients were given a symptom questionnaire, a fluid intake and urine output measurement protocol and a urine test strip set for an additional 5 days after discharge. Patients already documented their fluid intake and urine output during the postoperative inpatient phase and continued to do so for every single total of five days after discharge. Urine output was assessed using a milliliter measuring cup. Urine specific gravity was assessed on each of the five days after discharge by the patients using the urine test strip (Multistix^®^ 10SG; Bayer AG, Leverkusen, Germany) twice a day. Patients were also instructed to document their bodyweight unadorned twice a day (at 08:00 a.m. and 08:00 p.m. during the five-day period after discharge). The results were to be documented in a standardized questionnaire that was developed by endocrinologists and neurosurgeons of our university hospital as an approach to improve the detection of postoperative hyponatremia (Supplementary Materials). The questionnaire included a symptom catalogue, including symptoms like cephalgia, nausea, emesis, vertigo, loss of appetite, concentration difficulties and disorientation (see Supplementary Materials). In the case of abnormalities or clinical symptoms, the patients were told to immediately seek medical advice. DSH was defined as a reduction in the plasma sodium concentration to <135 mmol/L following inpatient discharge with concurrent clinical symptoms. The degree of hyponatremia was categorized to mild (plasma sodium concentration of 130–134 mmol/L), moderate (125–129 mmol/L), or severe (<125 mmol/L) [20]. The in-vitro diagnostic A-LYTE^®^ Integrated Multisensor was used for the quantitative measurement of sodium concentrations. As we did not measure serum osmolality, urine sodium concentration or urine osmolality after hospital discharge, we were not able to differentiate SIADH from postoperative hyponatremia. Management of hyponatremia was based on plasma sodium concentrations and degree of symptoms of the patients. Study inclusion criteria were age over or equal to 18 years, transsphenoidal pituitary surgery and the ability of giving written informed consent. Study exclusion criteria were the age of under 18 years, the diagnosis of syndrome of inappropriate antidiuretic hormone secretion (SIADH) or Diabetes Insipidus (DI) until the day of discharge, the use of vaptans, inability to measure weight post-discharge, drug addiction and refusal of participation. Patients sent the forms back via mail, were contacted via telephone in the occurrence of abnormalities and were consequently asked about postoperative complications. While we recommended to seek medical advice in the case of symptoms or clinical abnormalities at our university hospital, re-presentation in our clinic in the case of symptoms was not a requirement for study participation. The participants had free choice of doctor for the clarification of possible hyponatremia.

### 2.4. Statistical Analysis

Statistical analyses were performed using SPSS Version 27 and figures were created using Graph Pad Prism Version 8.0 (GraphPad Software, Inc., San Diego, CA, USA). The level of statistical significance was set at a *p* value of <0.05. Continuous data are presented as mean ± SD; categorical and nominal data are presented as frequency and percentage. Demographic and preoperative characteristics were recorded for individual patients. Fisher exact tests and Student *t*-tests were used to interrogate individual characteristics that might be associated with postoperative hyponatremia. For the analysis and comparison of bodyweight changes, fluid balance and urine specific gravity within both groups, we considered the changes within the two days before the event of hyponatremia (denoted as day −2 and day −1) and the day of the event of hyponatremia (denoted as day 0).

## 3. Results

### 3.1. Study Flow Chart

**Figure 1:**
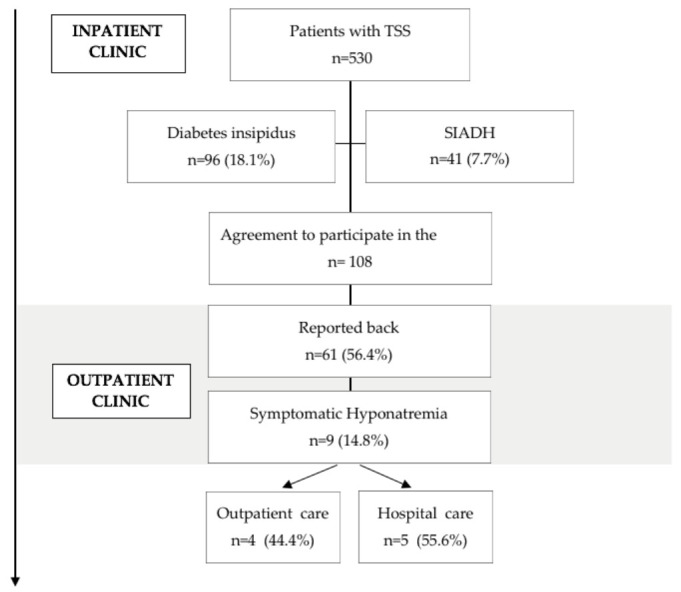
Flow chart of patient enrolment.

A total of 530 patients were operated for suprasellar and sellar pathologies at the Department of Neurosurgery of the University Hospital Hamburg-Eppendorf, all using an endoscopic transsphenoidal approach. A flow chart of patient enrolment is shown in Figure 1.

### 3.2. Patient and Tumour Characteristics

The selected demographic and clinical characteristics of enrolled patients with and without hyponatremia are shown in Table 1. Of the sixty-one patients (56.5%) who returned the questionnaire for analysis, thirty-nine of them (63.9%) were female and twenty-two (36.1%) were male. The mean age was 50.5 ± 15.5 years (range 14–84). The average inpatient postoperative stay of all 61 patients was 5 ± 2 days (median: 5 days, range 3–17). The most commonly operated pituitary pathology was adenoma (*n* = 28 functioning adenomas, *n* = 25 non-functioning adenomas). Functioning adenomas included Prolactinoma (*n* = 7), Cushing’s Disease (*n* = 12), Acromegaly (*n* = 8) and TSH-oma (*n* = 1). Further pathologies included Rathke’s cleft cyst (*n* = 2), Arachnoid Cyst (*n* = 1), Chordoma (*n* = 1), Meningioma (*n* = 1), Cavernous Hemangioma (*n* = 1), and Pituicytoma (*n* = 1). From a surgical perspective, gross total resection (GTR) was possible in 52 cases (85%), and intended partial resection (PR) in eight cases (13%). The Arachnoid Cyst was successfully fenestrated (2%).

### 3.3. Postoperative Hyponatremia after Hospital Discharge

**Figure 2:**
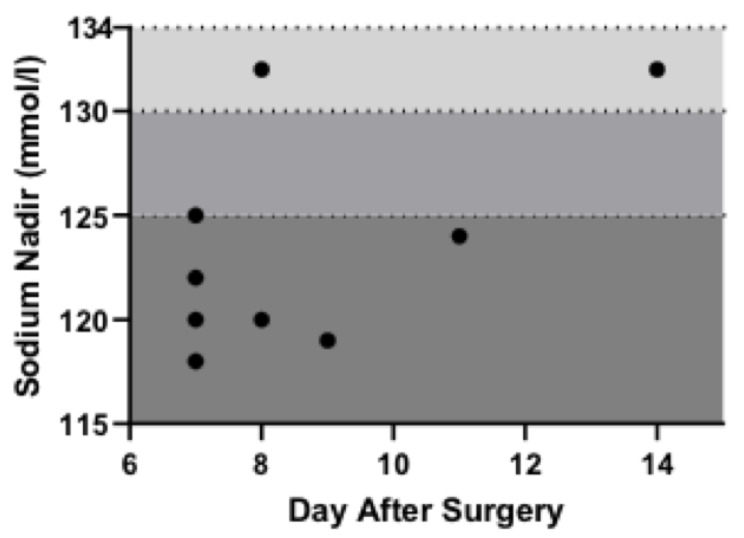
Date and severity of DSH.

Of the 61 out of 530 patients included in this study for further analysis, nine patients (14.8%) were diagnosed with delayed, symptomatic hyponatremia after hospital discharge. Mild hyponatremia occurred in two patients (2/61; 3.3%), moderate hyponatremia in one patient (1/61; 1.6%) and severe hyponatremia was reported in six patients (6/61; 9.8%). There was no significant difference in the average postoperative inpatient stay between patients with vs. without hyponatremia (5 ± 1 vs. 5 ± 2 days, *p* > 0.99). Hyponatremia occurred on average 8.6 postoperative days after surgery (median time: 8 days, range 7–14). The date and severity of DSH is shown in Figure 2. The mean plasma sodium concentration of the DSH cohort was 123 mmol/L (range 119–132 mmol/L). Patients with and without DSH showed no significant differences in plasma sodium levels at POD 1 (Table 1). After the diagnosis of hyponatremia was made, based on clinical symptoms with a consecutive medical presentation of the patients, four patients (4/9; 44.4%) received outpatient care post-surgery, two (2/9; 22.2%) were treated in a regular hospital ward and three (3/9; 33.3%) required intensive care treatment (Appendix A). The use of vaptans occurred in three patients (3/9; 33.3%) within the treatment after diagnosis of DSH. As the use of vaptans is only licensed for SIADH management, the diagnosis of SIADH was made in the outpatient setting. Three patients (3/9; 33.3%) received sodium infusions or sodium chloride tablets and three patients (3/9; 33.3%) were treated through fluid restriction. All nine patients with hyponatremia made a full recovery without significant complications. There was no associated mortality. The patient who received sodium chloride tablets was treated by his general practitioner.

### 3.4. Factors Associated with DSH

**Figure 3:**
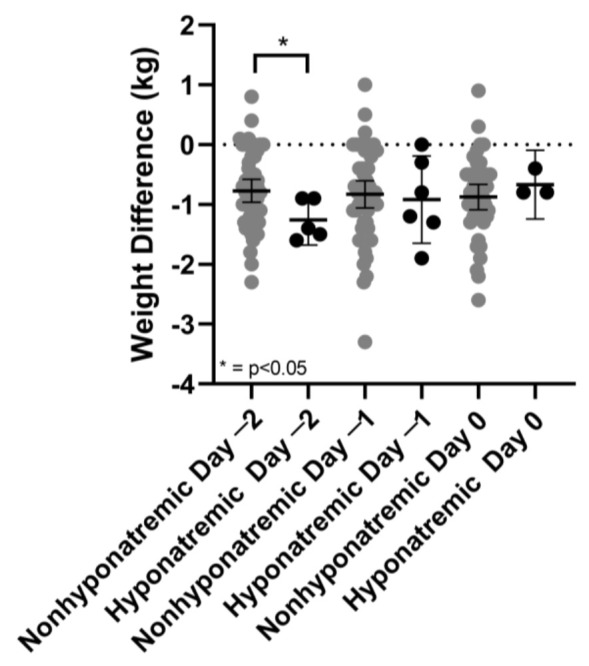
Weight differences of hyponatremic (*black*) vs. nonhyponatremic (*grey*) patients 2 days before (day −2), 1 day before (day −1) and on the day of event of hyponatremia (day 0), Error Bars represent mean with 95% CI, * = *p* < 0.05 student’s *t*-test.

All patients with hyponatremia were female (male: 0/22 vs. female: 9/39 (23.1%, OR 0.3 (*p* = 0.02)), *p* = 0.015) and patients affected by DSH had a significantly lower body mass index (BMI) compared to patients without hyponatremia (BMI 24.3 ± 2.8 kg/m^2^ vs. 27.5 ± 5.1 kg/m^2^; *p* = 0.02). The association between lower BMI in the hyponatremia group persisted when comparing only female sex in both groups, albeit the results only approached statistical significance, probably due to the small hyponatremia cohort (BMI: 24.26 kg/m^2^ ± 2.76 vs. 26.70 kg/m^2^ ± 5.48 kg/m^2^, *p* = 0.087). Age and tumour size did not show a significant association with the manifestation of hyponatremia (Table 1), neither did the type of resection (GTR: 45/52 vs. 7/9; PR: 6/52 vs. 2/9, *p* = 0.634). Two days before the event of hyponatremia with a median of six days after surgery, postoperative body weight delta between morning and afternoon weight was significantly different between groups (hyponatremia 1.26 kg (*n* = 5) vs. no hyponatremia 0.79 kg (*n* = 52), *p* < 0.05; Figure 3). All other timepoints were not significantly different. Univariate analysis using students’ *t*-test of the whole cohort did reveal differences for fluid output two days before the event of hyponatremia with decreased urine outputs in hyponatremic patients, but these findings were not significant. There were also no significant changes in fluid uptake in both groups, neither were there respective differences between day −2 and day −1 or day −2 and the day of event of DSH. There were also no statistically significant differences in morning and afternoon specific urine weight or significant differences concerning the difference between both these variables for each day.

### 3.5. Clinical Symptoms of DSH

**Figure 4:**
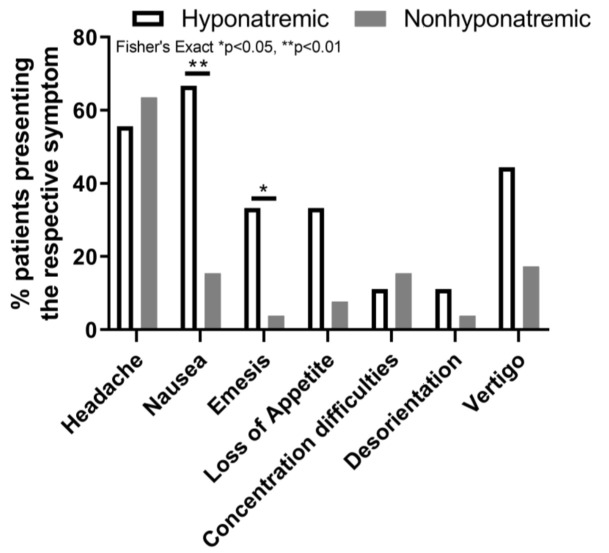
Frequency distribution of clinical symptoms which presented between day one–five after discharge.

Of all patients, 55.7% (34/60, one missing) showed symptoms on day one after hospital discharge, 54.1% (33/60) on day two, 55.7% (34/57) on day three, 63.9% (39/57) on day four and 65.6% (40/55) on day five after discharge. Patients with hyponatremia presented significantly more nausea 66.7% (6/9) vs. 15.4% (8/52) (OR, Fisher’s Exact, 11.4; *p* < 0.01) and emesis 33.3% (3/9) vs. 3.8% (2/52) (OR 12.5, Fisher’s Exact, *p* < 0.05) during day one–five (Figure 4). Concerning appetite loss, 33.3% (3/9) of patients with hyponatremia compared to 7.7% (4/52) in the non-hyponatremia group were affected, with results approaching statistical significance (*p* = 0.059). Similarly, for vertigo with 44.4% (4/9) vs. 17.3% (9/52), *p* = 0.09. The occurrence of concentration difficulties (1/9 vs. 8/52) and headaches (5/9 vs. 33/52) were not statistically different between both groups.

### 3.6. Correlation Analysis

A spearman correlation analysis of all recorded parameters with hyponatremia showed significant correlations for female sex (Rho = 0.275, *p* = 0.04) only. Individual weight changes between morning and afternoon weight did not show a significant correlation with the occurrence of hyponatremia (Day −2 Rho −0.041, *p* = 0.759, Day −1 Rho 0.071, *p* = 0.597, Day 0 Rho = 0.154, *p* = 0.248). Daily fluid input and output changes were not significantly correlated with hyponatremia (Day −2 Rho 0.159, *p* = 0.261, Day −1 Rho 0.115, *p* = 0.415, Rho 0.95, *p* = 0.496), neither were urine volumes (Day −2 Rho −0.013, *p* = 0.922, Day −1 Rho =−0.016, *p* = 0.908, Day 0 Rho = −0.151, *p* = 0.285).

## 4. Discussion

In this prospective, single-center study we evaluated screening parameters and searched for associated factors for DSH after TSS. We found that symptoms of nausea and emesis were associated screening parameters for DSH after clinical discharge, especially in women with lower BMI. There was a significant trend in the hyponatremic group regarding postoperative weight changes between morning and afternoon weight, two days before the event of hyponatremia (1.26 kg (*n* = 5) vs. 0.79 kg (*n* = 52), *p* < 0.05).

We report a prevalence of DSH of 14.8%. This is a higher prevalence than reported in other studies with event rates ranging from 4%–12% [2,17,21,22]. The differences in prevalence could be explained by the additional symptom questionnaire that was handed out in our study cohort. This could have led to a lower patient threshold for reporting symptoms of hyponatremia and highlights the impact of handing out a symptom questionnaire after clinical discharge in terms of prompting patients’ awareness of hyponatremia. Additionally, predefined postoperative sodium controls might have led to an under-detection of hyponatremia in other reported studies. The prevalence of severe hyponatremia (<125 mmol/L) in our study cohort was 9.8% and is higher than the reported prevalence in other studies ranging from 3.8%–7.7% [2,17,23,24]. As we only included patients with symptomatic hyponatremia, who typically present with more severe forms of hyponatremia, this might have influenced the prevalence rate [17,23]. Hyponatremia occurred on average 8.6 days after surgery with a median of eight days (range 7–14). This finding is consistent with other studies [22,23]. After a median of six postoperative days (range 5–12), two days before the event of hyponatremia, the body weight delta between morning and afternoon weight was significantly different between both groups (*p* < 0.05). From a pathophysiological point of view, we know that arginine vasopressin (AVP) regulates the expression of the aquaporine water channel 2 in the kidney [25]. There is a daily variation in the plasma concentrations of AVP in healthy adults, demonstrating a nadir in the late afternoon [26]. Inappropriate AVP secretion initiated by pituitary damage that produces AVP secretion after TSS is the predominant mechanism for hyponatremia [27]. It is associated with slight weight gain of 5%–10% of body weight [28]. A delayed overexpression of vasopressin after TSS [29], independent of the type of resection, may have led to a dysregulation of the circadian rhythm in our study cohort. This might have resulted in significant weight changes before the manifestation of hyponatremia. However, the small number of hyponatremic patients with available morning and afternoon weights and the overlap of weight delta between the non-hyponatremic and hyponatremic group, makes it difficult to conclude that postoperative body weight delta between morning and afternoon weight is an associated factor for DSH. Nevertheless, we report a trend.

Consistent with body weight changes between morning and afternoon weight in the hyponatremic group, two days before the event of hyponatremia, we measured a negative daily fluid balance with a decreased output in the hyponatremic group, but this finding was not significant. One reason could be the small patient cohort. Another reason might be, that although measuring fluid input and output is a simple procedure, we know that in practice, recording fluid intake [30] and output [31] is easily affected by human errors and can lead to inadequate and invalid fluid measurements.

Looking at the clinical symptoms of our study cohort, the overall prevalence of symptoms from day one to day five after hospital discharge increased in both groups, with 55.7% (34/60, 1 missing) showing symptoms on day one after hospital discharge to 65.6% (40/55) presenting with symptoms on day five after hospital discharge. This finding could have been biased by the study design, which included a symptom questionnaire and might have led to a report bias. We found nausea and emesis being significantly associated with DSH. In addition to the symptoms of DSH, there are currently several other markers associated with a high risk of postoperative hyponatremia. As previously described [2,17], we found female sex and a lower BMI to be an associated factor for DSH. All patients but one were older than 50 years, which has been described before by Tomita et al. 2019. [5] Another factor that was described to favor delayed hyponatremia is tumour size [32], which was not associated with statistical significance in our study cohort.

The state-of-the-art treatment of DSH depends mainly on the severity and duration of hyponatremia. The cut-off point for distinguishing between acute and chronic hyponatremia is 48 h. Unfortunately, in clinical practice, the distinction between acute and chronic hyponatremia is often unclear because it is usually, as in our cohort, not known when the drop in serum sodium concentration started. When classification of hyponatremia as acute or chronic is not possible, the European Society of Endocrinology (ESE) [20] recommends hyponatremia to be considered chronic unless there are reasons to believe it is acute. In cases of moderate (nausea, headache, malaise, confusion) or severe (vomiting, somnolence, coma) symptoms, the risk of cerebral oedema predominates and immediate treatment is indicated in these cases, regardless of the biochemical degree or timing (acute or chronic) of hyponatremia. If possible, conditions or medications that may promote or cause hyponatremia should be ruled out or discontinued. One patient with DSH in our cohort was taking hydrochlorothiazide but was already doing so before surgery.

If symptoms are severe, inpatient monitoring is indicated. Immediate treatment with a single i.v. infusion of 150 mL of 3% hypertonic saline or equivalent for 20 min should be given and repeated if this does not result in an increase in serum sodium concentration of 5 mmol/L (for moderate symptoms within 24 h). Serum sodium concentration should be limited to an increase of 10 mmol/L in the first 24 h and 8 mmol/L in the following 24 h to avoid osmotic demyelination syndrome. Serum sodium concentrations should be checked at 1, 6 and 12 h following infusion.

The guideline development group of the ESE recommends fluid restriction as first-line treatment for the diagnosis of SIADH. Vasopressin receptor antagonists are not recommended because of the increased risk of correcting hyponatremia too quickly. In our study group, thee patients with DSH received fluid restriction, another two patients received sodium infusions and one patient received sodium chloride tablets by his general practitioner. The vasopressin receptor antagonist *Tolvaptan* was given to three patients with DSH in a dosage of 3 mg each. This dosage is significantly lower than the usual dosages of 15 mg to 60 mg per day and was a composite formulation for further titration. We made good experience in our clinical practice with using doses of 3 mg Tolvaptan for the treatment of DSH and the literature supports this approach [33]. However, it has to be stated that it is currently not recommended by the guidelines.

To date, there are several approaches for the prevention of postoperative hyponatremia. Lee et al., recommend regular monitoring of sodium concentrations for all patients who underwent TSS for up to two weeks after TSS [23]. This might be a challenge for functionally disabled patients. Also, patients living in rural areas with a shortage of general practitioners might not be able to monitor their sodium levels. From an economic point of view, handing out a patient questionnaire is much more attractive for our healthcare systems. Looking into the future, such questionnaires could also be digitalised and thus further simplify detection of DSH for patients and their physicians. A growing body of literature supports mild fluid restriction during the early postoperative TSS period to prevent hyponatremia and decrease the risk of readmission [4,34]. Since adequate compliance is mandatory and patients suffering from comorbidities might need to be excluded, this approach is only feasible for a few patients. As DSH is a serious complication after TSS, there is no “one-fits-all” solution for its prevention. By providing patients with a symptom questionnaire, their awareness is raised. Subsequently, their doctors will also be sensitised when their patients present with symptoms of DSH at the respective practices or hospitals. Therefore, our findings can be considered as an additional, practical and cost-effective approach to detect DSH. We recommend two main measures: 1. Providing patients with a symptom questionnaire at the time of discharge and 2. performing twice-daily body weight measurements for eight days after surgery.

### 4.1. Limitations

This study has several limitations. First, due to missing continuous sodium measurements after discharge, asymptomatic patients with hyponatremia could have been undiagnosed. Second, the baseline difference in the group characteristics with a small patient cohort with hyponatremia makes a statistical conclusion difficult. Finally, there may have been other variables not collected in this database that may have contributed to the development of DSH.

### 4.2. Conclusions

Taken together, diagnosis of DSH in the outpatient setting remains challenging. Secondary adrenal insufficiency and diuretic use must be ruled out. We detected a relevant number of patients with DSH after hospital discharge. Markers associated with increased risk for hyponatremia were female gender and lower BMI. Nausea and emesis seem to be associated symptoms for hyponatremia and the use of a symptom questionnaire could be an easy and feasible approach for the detection of delayed, symptomatic hyponatremia after transsphenoidal surgery. We report a significant trend in weight changes between the morning and afternoon weight in the hyponatremic group two days before the event of hyponatremia. Due to our small patient cohort, further research is needed to draw conclusions from this trend.

## Figures and Tables

**Table 1:** Patient demographic and clinical characteristics.

Characteristics	Hyponatremic	Nonhyponatremic	*p*-Value
No. of patients	9	52	
Age (years)	57 ± 10	50 ± 15	0.11
Female (%)	9 (100%)	39 (75%)	**0.02**
BMI (kg/m^2^)	24.26 (±2.8)	27.45 (±5.13)	**0.02**
History of hypertension (%)	4 (44.4%)	17 (32.7%)	0.71
History of cardiovascular disease (%)	1 (11.1%)	2 (3.8%)	0.39
History of Diabetes mellitus (%)	2 (22.2%)	4 (7.7%)	0.21
History of thyroid disease (%)	2 (22.2%)	16 (30,8%)	0.71
Nonfunctioning adenoma (%)	5 (55.5%)	20 (38.4%)	NA
Prolactinoma (%)	1 (11.1%)	6 (11.5%	NA
Cushing disease (%)	1 (11.1%)	11 (21.1%)	NA
Acromegaly (%)	2 (22.2%)	6 (11.5%)	NA
TSHoma	0	1 (1.9%)	NA
Rathke cleft cyst	0	2 (3.8%)	NA
Chordoma	0	1 (1.9%)	NA
Pituicytoma	0	1 (1.9%)	NA
Arachnoidal cyst	0	1 (1.9%)	NA
Meningeoma	0	1 (1.9%)	NA
Cavernous Hemangioma	0	1 (1.9%)	NA
Not classified Adenoma	0	1 (1.9%)	NA
Tumor size (cm)	1.9 × 1.5 × 1.5	1.5 × 1.2 × 1.5	NA
Coronal	1.98 ± 0.79	1.50 ± 0.78	0.12
Sagittal	1.53 ± 0.54	1.27 ± 0.62	0.21
Cranio-Caudal	1.58 ± 0.64	1.49 ± 0.94	0.70
Serum sodium on POD 1 (mmol/L)	139 ± 3	141 ± 2	0.19

Data are shown in mean ± SD and relative frequency. Abbreviations: NO. = number; NA = not assessed; BMI = Body Mass Index; POD = Postoperative Day.

## Data Availability

The data that support the findings of this study are available from the corresponding author upon reasonable request.

## Supplementary Materials

The following supporting information can be downloaded at: https://www.mdpi.com/article/10.3390/jcm12010306/s1, Patient Questionnaire.

## Appendix A. Severity, Occurrence and Therapy of the 9 Patients with DSH

**Patient No.**	**Sodium Nadir**	**POD**	**IP** **Intensive Care Unit (ICU)** **Outpatient (OP)**	**Duration of Hospital Stay**	**Therapy**
1	122	7	ICU	7	Sodium infusion
2	118	7	ICU	5	Sodium infusions and Prednisolone
3	132	14	OP	-	Fluid restriction
4	120	8	IP	6	multiple doses 3 mg Tolvaptan
5	120	7	ICU	6	multiple doses 3 mg Tolvaptan
6	124	11	IP	2	single dose 3 mg Tolvaptan
7	125	7	OP	-	Sodium pills
8	119	9	OP	-	Fluid restriction
9	132	8	OP	-	Fluid restriction
