# Supplementary figures and images for: The Potential of Self-Assessment and Associated Factors for Delayed Symptomatic Hyponatremia Following Transsphenoidal Surgery: A Single Center Experience
Source: J Clin Med. 2022 Dec 30;12(1):306. doi: 10.3390/jcm12010306 (PMC9821286; doi:10.3390/jcm12010306)

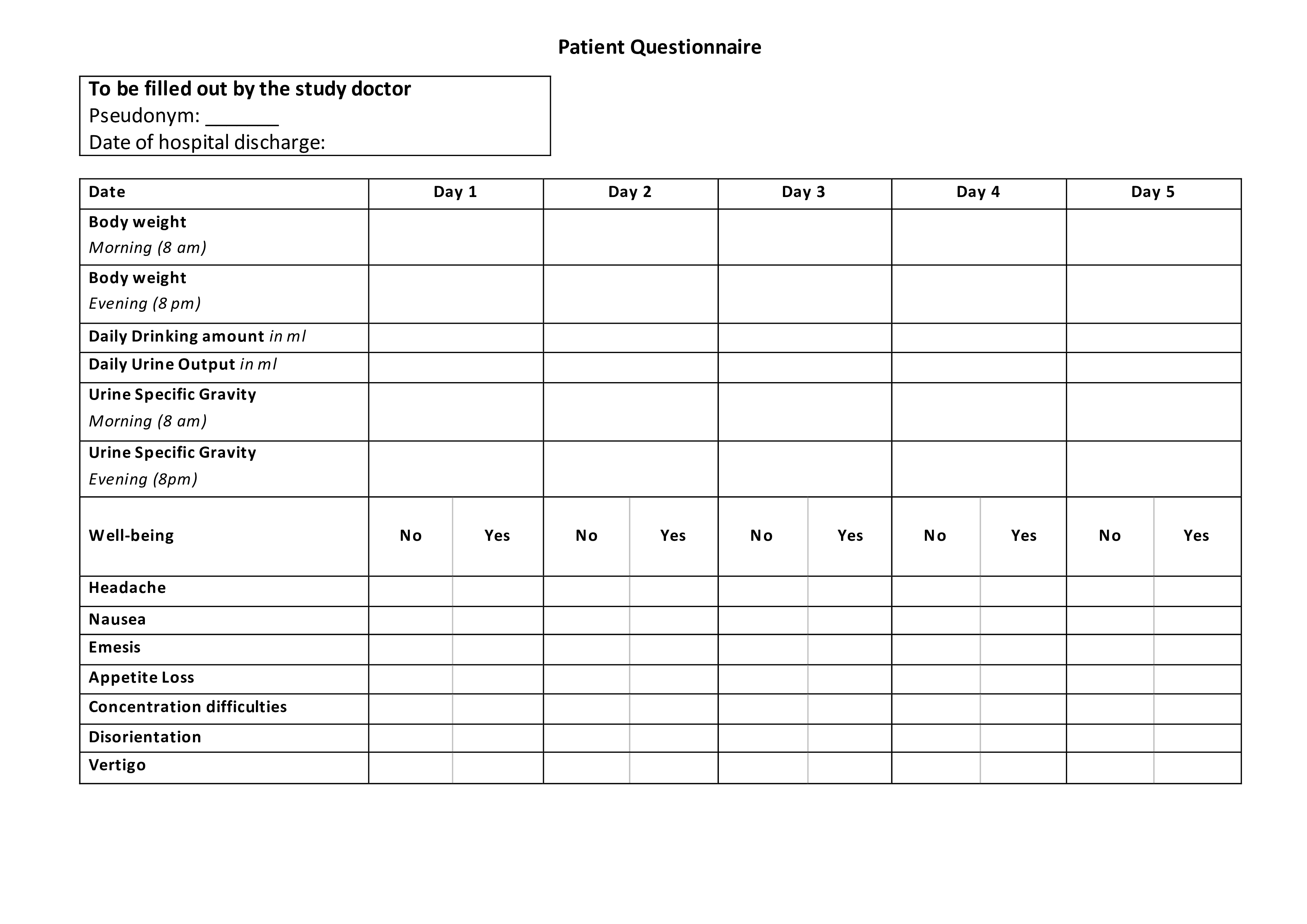

Supplement: Supplementary file 1 [file jcm-12-00306-s001.zip › Supplementary Material .jpg]
